# Microstructure and Deformation of Coronary Stents from CoCr-Alloys with Different Designs

**DOI:** 10.3390/ma8052467

**Published:** 2015-05-08

**Authors:** Sabine Weiss, Bojan Mitevski

**Affiliations:** Brandenburg University of Technology Cottbus-Senftenberg, Konrad-Wachsmann-Allee 17, Cottbus 03046, Germany; E-Mail: bojan.mitevski@uni-due.de

**Keywords:** coronary stents, design, strut thickness, L-605, F-562, mechanical behavior, microstructure, orientation mapping, dislocation structure

## Abstract

Coronary heart disease is still one of the most common sources for death in western industrial countries. Since 1986, a metal vessel scaffold (stent) has been inserted to prevent the vessel wall from collapsing. Most of these coronary stents are made from CrNiMo­steel (316L). Due to its austenitic structure, the material shows a good combination of strength, ductility, corrosion resistance, and biocompatibility. However, this material has some disadvantages like its non-MRI compatibility and its poor fluoroscopic visibility. Other typically used materials are the Co­Base alloys L-605 and F-562 which are MRI compatible as well as radiopaque. Another interesting fact is their excellent radial strength and therefore the ability to produce extra thin struts with increased strength. However, because of a strut diameter much less than 100 μm, the cross section consists of about 5 to 10 crystal grains (oligo­crystalline). Thus, very few or even just one grain can be responsible for the success or failure of the whole stent. To investigate the relation between microstructure, mechanical factors and stent design, commercially available Cobalt-Chromium stents were investigated with focus on distinct inhomogeneous plastic deformation due to crimping and dilation. A characteristic, material related deformation behavior with predominantly primary slip was identified to be responsible for the special properties of CoCr stents.

## 1. Introduction

Most commercially available coronary stents are made of 316L type CrNiMo­steels (e.g., DIN EN 1.4441) [1]. Due to its austenitic structure, the material shows a good combination of strength, ductility, corrosion resistance, and biocompatibility [2,3,4]. However, this material has some disadvantages like its non-MRI compatibility and its poor fluoroscopic visibility [5]. Other typically used materials are the Co­Base alloys L-605 and F-562 which are MRI compatible [6] as well as radiopaque [7]. Another interesting fact is their excellent radial strength, flexibility and deliverability and therefore the ability to produce extra thin struts with increased strength [5,8]. Stent strut thickness plays an important role in vascular injury and consequent neointimal proliferation. Clinical studies revealed that the thin-strut stents are significantly associated with reduced early restenosis [9,10,11]. A positive influence of thin strut stents on long-term luminal response with the results that neointimal atherosclerotic change occurred more frequently for patients with thick-strut stents and that the incidence of late in stent restenosis was significantly lower in the thin-strut group of patients was observed, too [7,12,13]. However, thin strut stents especially from CoCr-alloys are not indisputable. In recent investigations significantly higher acute elastic recoil was observed for thin strut CoCr-stents [14]. The authors compared 316L steel stents with a strut thickness of 135 μm, PtCr stents and CoCr stents, both with strut thicknesses of 81 μm. Low acute recoil was found for 316L and PtCr in contrast to much higher recoil for CoCr. Because of the same strut thickness of the PtCr stents and the CoCo stents they assume, that acute stent recoil is more correlated with stent design and materials than with stent strut thickness. Another current study used nanoindentation to analyze the local mechanical properties of stents. They could identify regions of high hardness and related them to the gradient of plastic strains generated during the deployment process and the associated stress. These regions were correlated with the locations where macroscopic fractures have been observed in both mechanically tested and human explanted stents because of a reduction in plastic work ratio [15].

Despite all these investigations there is only limited knowledge available about the correlations between plastic deformation, strut thickness, design, material and recoil. Furthermore, due to the small dimensions of stents, the material has an oligo­ crystalline structure (only a few grains distributed over the cross section of a stent strut). These structures can in fact neither be described as multi­crystalline materials, nor be treated as single crystals. The result is an orientation dependent inhomogeneous deformation behavior [16,17]. With regard to the importance of the orientation parameter, the Electron BackScatter Diffraction (EBSD) technique has been used to compare the crystallographic orientation of the grains in CoCr stents with different designs after deformation due to dilation. By means of transmission electron microscopy (TEM) the deformation mechanisms can be investigated. For this study, the microstructural alterations after plastic deformation of three different stent designs (Multi Link Vision™, GUIDANT Corporation, Santa Clara, CA, USA; Coroflex^®^ Blue, B. Braun Melsungen AG, Melsungen, Germany and Driver Medtronic Inc., Minneapolis, MN, USA) were investigated.

## 2. Experimental

### 2.1. Material

**Figure 1:**
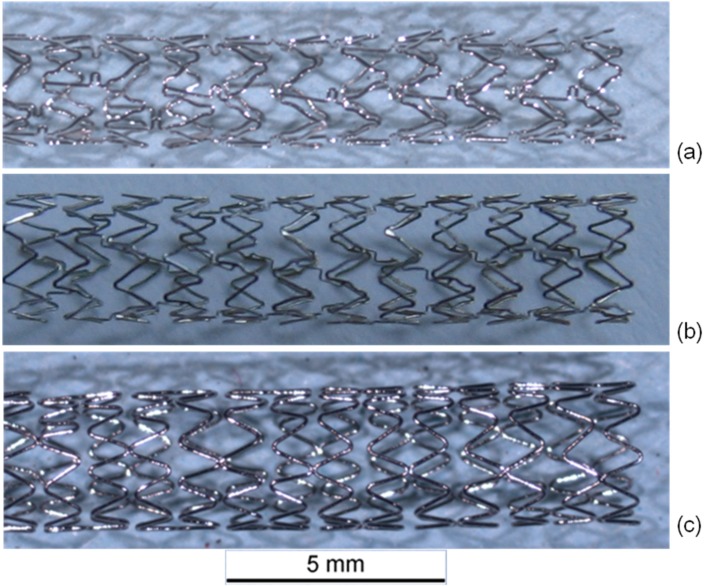
(**a**) Multi Link Vision (L-605, 81 μm); (**b**) Coroflex^®^ Blue (L-605, 60 μm); (**c**) Medtronic Driver (F-562, 91 μm).

For this purpose, commercially available cobalt chromium based L-605 and F-562 coronary artery stents of three different designs (Multi Link Vision L-605, Coroflex^®^ Blue L-605 and Driver F-562) with characteristic strut thicknesses were compared. Multi Link Vision and Driver are among the market leading cobalt chromium stents. Coroflex^®^ Blue was chosen because of its very low strut thickness. The stent designs are depicted in Figure 1.

All three designs are laser cut slotted tube stents with typical v-shape structure. The Driver design exclusively consists of regular v-shapes with 91 μm strut thickness. In the Coroflex^®^ Blue with only 60 μm strut thickness one of the two flanks is curved and in the Multi Link Vision design with 81 μm strut thickness stabilization struts with loops connecting two lines of the v-structure are added.

### 2.2. Preparation

All stents were dilated in air according to the descriptions in the manufacturers’ instructions. Microstructure characterization of dilated stents was carried out by means of scanning electron microscopy (SEM) as well as transmission electron microscopy (TEM). Furthermore, single grain orientation determination by means of Electron BackScatter Diffraction (EBSD) was performed, in order to reveal microstructural alterations during deformation. For some stents no further metallographic preparation was necessary to obtain EBSD pattern, because the final stent production step is electrochemical polishing. Stents with a coated surface were electrochemically polished for 10 s in an electrolyte composed of sulfuric acid and phosphorous acid to reach the bare metal surface.

### 2.3. SEM-Imaging and EBSD-Measurement

For measurement some geometrical difficulties had to be taken into account; Because of the tube profiles of the stents significant decrease in pattern quality is observed depending on the position on the sample surface. For a tube with 3 mm diameter, very close to the top of the tube in the secondary-electron (SE)-image, a small part of the upper region of the EBSD-pattern is shaded and appears dark. Best pattern quality is obtained in a distance of about 50 μm from the top; at about 100 μm from the top the shaded area in the lower part of the pattern has increased substantially and at a distance of about 200 μm (from the top) no orientation determination was possible any more. The measurable distance depends on the radius R of the tube (or stent) and can be calculated as:

*u* = R (1 − cos α) (1)

α corresponds to 90° minus the minimum tilting angle at which pattern can be reached (for traditional EBSD equipment α is between 65° and 75°). Further information on this field is published elsewhere (by the authors) [18].

For SEM images a TESCAN MIRA\\ (TESCAN, Brno, Czech Republic) was used. An SEM Gemini 1530 (Zeiss, Oberkochen, Germany) with an EBSD system Crystal (Oxford Instruments, Wiesbaden, Germany) and calculation software Channel 5 from HKL­Technology (Hobro, Denmark) as well as an EBSD system from EDAX (Tilburg, The Netherlands) with OIM™ 6.0 Software were used. EBSD analyzes were carried out by two different people using two different EBSD systems without knowledge about the results of the respective partner to support the repeatability of the results. For both an accelerating voltage of 20 kV and a working distance of 15 mm were applied to obtain single grain orientations as well as orientation maps. The orientation differences are presented by means of Kernel misorientation maps.

### 2.4. TEM-Imaging

For the investigation of stents by means of TEM, parts of interest were cut out with focused ion beam technique for direct observation or with special preparation scissors for further manual preparation. For manual preparation these parts were inserted into a grid net. Stent and net were glued together using a suitable adhesive (M­Bond 610, Gatan GmbH, Munich, Germany). Afterwards it was ground down to a thickness of 80 μm and further thinned from both sides using a dimple grinder (Model 656, Gatan GmbH, Munich, Germany) and a nitrogen cooled ion mill (Model Pips II cool; Gatan GmbH). TEM investigations were performed with a Philips EM 400 TEM (FEI, Eindhoven, the Netherlands) as well as a Zeiss EM 912 (Zeiss, Oberkochen, Germany) both using an accelerating voltage of 120 kV. For TEM imaging the results of three different investigators as well as FIB or manually prepared specimens were analyzed. The images presented in Section 3 are representative for the materials microstructure of the corresponding CoCr stents.

## 3. Results and Discussion

**Figure 2:**
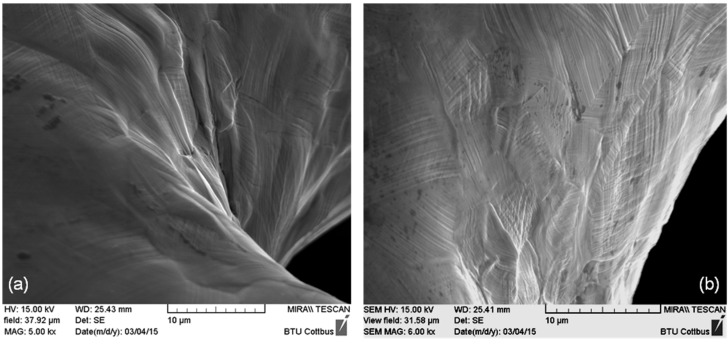
(**a**) Inner part of a stent bow and (**b**) passage between bow and strut of a Driver Stent (F-562) with slip traces on the surface as well as extrusions and intrusions of grains.

After dilation, stents of all three designs exhibit regions of high deformation with characteristic deformation structures like slip traces on the surface. In contrast to this, before dilation, a shiny polished surface was apparent. In Figure 2, representative SEM images of the inner part of stent bows of a Driver Stent are depicted. Slip traces (fine parallel lines) extrusions and intrusions of grains are clearly visible. This occurrence is generated by heavy plastic deformation and is accompanied with orientation gradients within the deformed grains. These so called misorientations are correlated to the amount of plastic deformation in the material.

**Figure 3:**
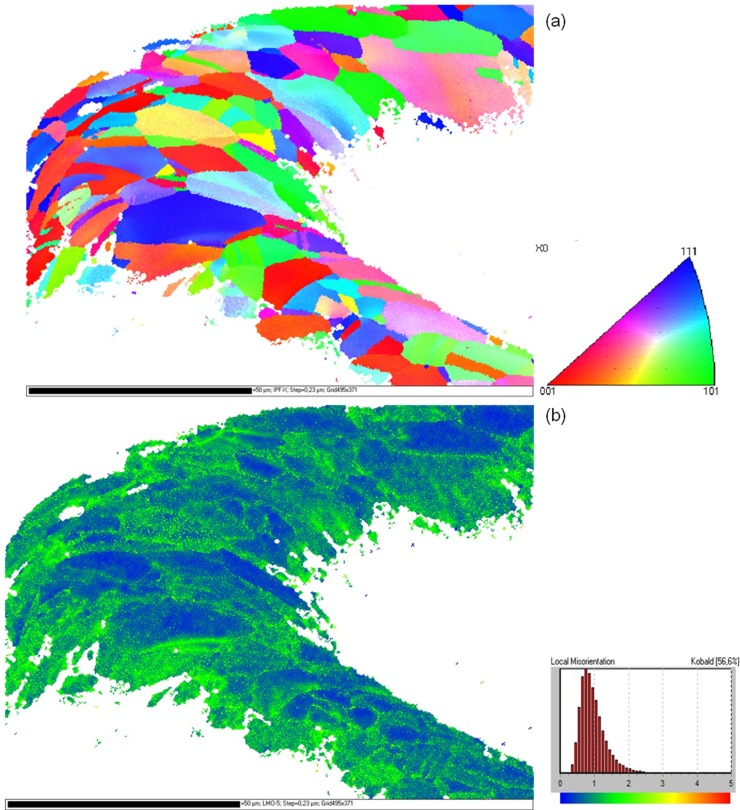
(**a**) Inverse pole figure orientation mapping of an F-562 stent bow (Driver) after dilation; (**b**) Kernel misorientation mapping of this stent bow. The colorings of the mappings are according to the given legends.

Orientation mappings of several stent bows of all three types of stents were measured. A typical grain orientation determination at a highly deformed stent bow of a Driver Stent after dilation is shown in Figure 3a. The orientation data is represented parallel to the longitudinal direction of the stent. The coloring of the mapping is in accordance to the legend in Figure 3a. As available from the mapping the material has an oligo-crystalline structure with only few grains distributed over the strut diameter. In contrast to the material prior to load [19] where completely homogeneous orientation mappings without any orientation gradient were measured, regions with large misorientations occur, identifiable from the iridescent colors within the grains. The high deformation becomes more obvious in the corresponding local misorientation map (Figure 3b). Low misorientations occur in blue and increasing misorientations are represented by green and yellow colors. A concentration of large orientation differences in the inner and outer region of the stent bow as well as close to the grain boundaries becomes obvious.

**Figure 4:**
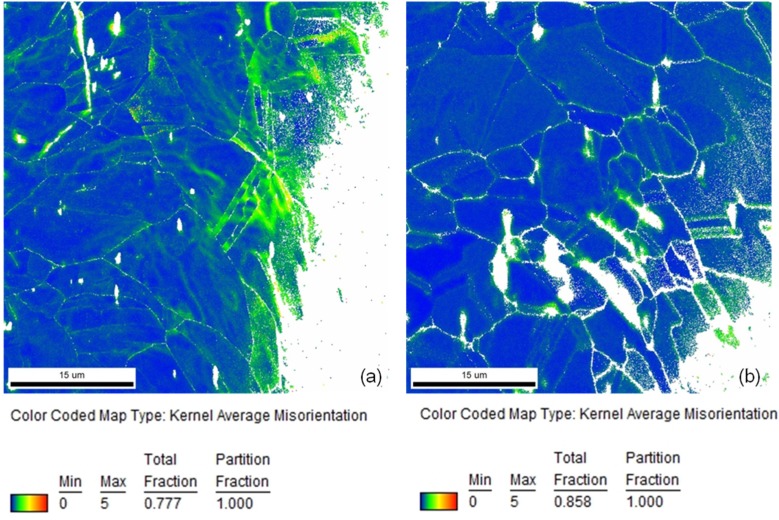
Comparison of local misorientation mappings of (**a**) a stent bow and (**b**) a stent strut of a Coroflex^®^ Blue Stent (coloring according to the legend).

In Figure 4a, a comparison of representative local misorientation mappings of a stent bow and a stent strut of a Coroflex^®^ Blue Stent is depicted. As available from the extended areas of green and yellow colors in the stent bow and in contrast to that predominantly blue coloring in the stent strut the much lower deformation in the strut than in the bow becomes obvious. This is in good agreement with the locations of high misorientations corresponding to high plastic deformation as well as high hardness measured by Kapnisis [15]. In his investigations of the hardness by means of nanoindentation he identified regions of high hardness in the stent bows and correlated them to high plastic deformation.

**Figure 5:**
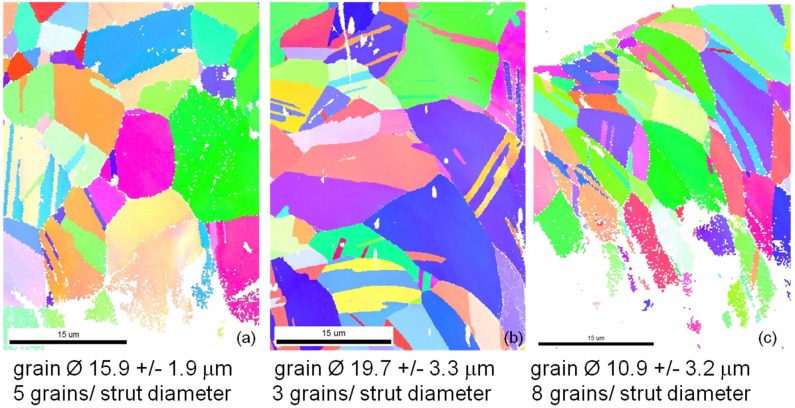
Inverse pole figure orientation mappings of (**a**) Multi Link Vision; (**b**) Coroflex^®^ Blue; and (**c**) Medtronic Driver stent. The colorings of the mappings are according to the legend in Figure 3a.

**Figure 6:**
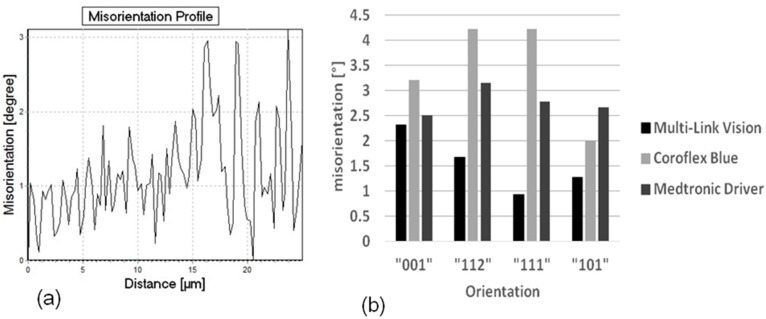
(**a**) Representative misorientation profile and (**b**) Maximum misorientation for different stent designs as a function of the grain orientation.

For comparison of the microstructures of the different stent designs in Figure 5 representative orientation mappings of bow segments of each stent type are given. All three designs show microstructures typical for CoCr alloys with relatively straight grain boundaries and lots of twins inside the grains but without preferred orientation. Regions with large misorientations occur in all three designs especially in the inner parts of the bows as available from more or less iridescent colors in the lower right parts of all mappings (Figure 5a–c). Excluding the twins the average grain diameters as well as the average number of grains per strut diameter have been determined. The lowest strut size to grain size ratio, thus, the strongest oligocrystallinity was measured for the Coroflex^®^ Blue, the largest for the Medtronic Driver. The influence of this ratio will be discussed later. It is difficult to present a quantitative comparison of the amount of misorientation by means of orientation mappings. Therefore the orientation gradient inside the grains along a line from one grain boundary to the opposite one was determined for a large number of grains (300 to 500 grains) separately for each stent design. One characteristic orientation gradient curve as well as the distribution of all orientation gradients of each stent design classified in four main crystallographic orientation groups is shown in Figure 6a,b, respectively.

A comparison between the three different designs indicates a significantly different behavior. The largest orientation gradients up to more than four degrees are observed in the Coroflex^®^ Blue stent design, the smallest, not more than 2.5 degrees, in the Multilink Vision. These results indicate that larger plastic deformation occurs in the bows of the Coroflex^®^ Blue stent design than in the Multilink Vision design. An interesting correlation with the results of Schmidt [20] is found: In his experiments he measured the elastic recoil of different stent designs and found the largest recoil for the Coroflex^®^ Blue stent design, medium recoil for the Driver, and lowest recoil for the Multilink Vision design. Therefore, one factor of this design dependence of the recoil is possibly attributed to the amount of plastic deformation in the material.

Furthermore the misorientation is found to be more or less dependent on the grain orientation (Figure 6b). In the Coroflex^®^ Blue and Driver stent designs the highest misorientations are measured in grains with {112}- and {111}- orientations. In the Multilink Vision design the highest misorientations are found in the {001} and {112} grains. This orientation dependence of plastic deformation is found to be more pronounced in the Coroflex^®^ Blue and in the Multilink Vision design and less pronounced in the Driver design. The characteristic strut thickness of Coroflex^®^ Blue is only 60 μm, combined with a relatively large grain size strong oligocrystallinity is obtained for this design. The Multilink Vision with 81 μm strut thickness and medium grain size shows oligocrystalline behavior, too. In contrast, the Medtronic Driver shows only low orientation dependency, thus a nearly polycrystalline behavior occurs. This result is in good correlation to the assumption that oligocrystalline deformation behavior begins when the grain size reaches the construction size. Up to now it is still not clear at which grain size to strut size ratio the homogenous deformation changes into an orientation dominated deformation but this value can be localized between 6 and 10 grains per diameter and seems to be floating. Nevertheless, the current results indicate that the influence of the individual grains on the total deformation decreases with increasing strut thickness and decreasing grain size. Hence, further investigations are necessary for a statistical validation of these results.

### Sliding Behavior of the Material

**Figure 7:**
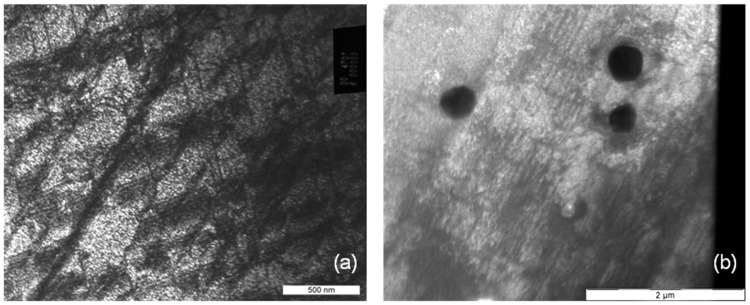
(**a**) Deformation structure and (**b**) nanocarbides in an L605 stent bow (Multilink Vision).

**Figure 8:**
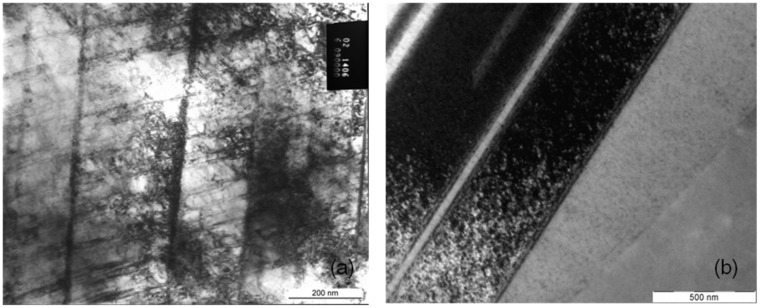
(**a**) Deformation structure and (**b**) deformation bands in an L605 stent bow (Coroflex^®^ Blue).

**Figure 9:**
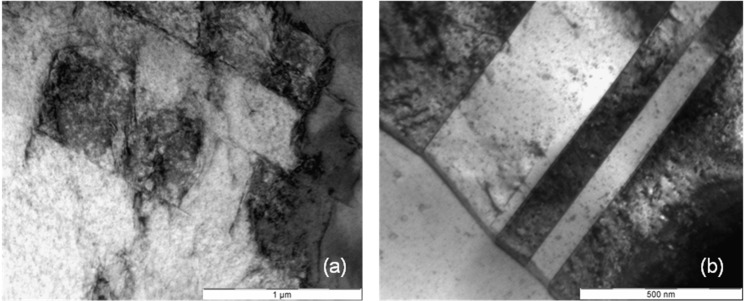
(**a**) Deformation structure and (**b**) deformation bands in an F-562 stent bow (Medtronic Driver).

As available from Figure 6a there is not only a high misorientation gradient within the deformed grains but also considerable fluctuations between neighboring measuring points, for example 1.5 degrees along a distance of only one micrometer. A possible explication is the typical deformation behavior of cobalt chromium alloys, the primary planar slip. Due to their low stacking fault energy slip is concentrated on only a few slip systems. There is nearly no possibility for the dislocations to deviate from their slip plane by cross slip or climbing. Figure 7, Figure 8 and Figure 9 present the microstructures of highly deformed areas of stent bows of all three designs. In all three designs characteristic deformation structures consisting of parallely­oriented and crossing deformation bands become visible. In addition, nanocarbides can be identified in the Multilink Vision microstructure (Figure 7b). A deformation structure like this can be an explanation for the considerable orientation fluctuations between neighboring measuring points observed in the orientation gradient distribution in Figure 6a. In Figures 8b and 9b two deformation bands are visible. The width of these bands, about 500 nanometers, is in exact correlation with the spacing of the oscillating orientation gradient. A deformation induced phase transformation from fcc alpha cobalt to hcp beta cobalt is possible.

Under planar slip dislocations, twins, stacking faults, and beta cobalt are generated and remain mobile, but only on their discrete sliding planes (see Figure 7a, Figure 8a and Figure 9a, straight lines crossing under distinct angles). Thus, this material shows such distinct localization of sliding under plastic deformation and fatigue, and is known for cyclic softening. However, such sliding behavior may be one reason for the higher tendency for elastic recoil in CoCr-alloy stents than in 316L stents. Even if the strength increases within the shear zone, the dislocations remain mobile but are concentrated on few slip systems with only few obstruction of dislocation movement due to immobile dislocations. Therefore back sliding of dislocations on their slip system can occur resulting in a decline of the plastic deformation introduced during dilation. As a result the “elastic” recoil is probably attributed to a plastic reaction of the material, too.

In summary, it appears that in those metals with predominantly primary slip mechanical instabilities, e.g., like shear bands, crack initiation and propagation take place at distinctly higher stress levels, but are concentrated on few slip planes which can be an explanation for the high radial stiffness of CoCr stents. The result of slip concentration is higher dislocation mobility and therefore a higher flexibility of the material, advantageous for delivery and dilation of the stent but combined with the negative effect of larger recoil. In the current study no macroscopic fractures have been observed and no cyclic load was investigated. However, materials with strongly localized slip like CoCr alloys tend to a moderate cyclic softening. Therefore, the potential risk of fracture can be classified as low. This estimation is in good agreement with the results of Kapnisis [15]. He compared stainless steel and CoCr stents with similar geometry with regard to their fracture behavior after 100 million load cycles. According to him, strut fracture could be attributed to fretting wear in overlapping regions in case of multiple stenting. For application of single CoCr stents in straight or low curved arteries an extremely low risk of fracture is expected, even lower than that of the market leading stainless steel stents.

## 4. Conclusions

The present study gives a more comprehensive understanding of the influence of the stent design on the structure property relationship under monotonic deformations, as a basis for ongoing development of new designs for stent optimization. Therefore, stents of different designs produced from the cobalt chromium alloys L-605 and F-562 were investigated. Microstructure characterization by means of scanning electron microscopy, as well as transmission electron microscopy and single grain orientation determination, show the microstructure and microtexture evolution during deformation. The comparison reveals differences in the amount of plastic deformation between the three designs. Larger plastic deformation occurs in the bows of the Coroflex^®^ Blue stent design than in the Multilink Vision design. Because of the small grain size/sample size ratio, orientation dependent deformation behavior occurs, which is more pronounced the thinner the strut thickness is.

The investigation of microstructure indicates that in all three CoCr alloy stent designs typical deformation behavior of primary planar slip takes place. This deformation behavior can be attributed not only to the high radial stiffness of CoCr stents but also to a high flexibility of the material, nonetheless combined with the negative effect of larger recoil.

As a first result, the advantageous properties of CoCr alloy stents predominate for clinical use, but the research is still ongoing. Further experiments, for example with regard to the grain size to strut size relationship of the stents or a modification of the dilation process are in progress and will be published in the near future.
